# Underfifty Women and Breast Cancer: Narrative Markers of Meaning-Making in Traumatic Experience

**DOI:** 10.3389/fpsyg.2019.00618

**Published:** 2019-03-26

**Authors:** Maria Luisa Martino, Daniela Lemmo, Anna Gargiulo, Daniela Barberio, Valentina Abate, Franca Avino, Raffaele Tortoriello

**Affiliations:** ^1^Department of Humanistic Studies, University of Naples Federico II, Naples, Italy; ^2^Clinical Psychology Unit, National Cancer Institute G. Pascale Foundation (IRCCS), Naples, Italy; ^3^Breast Surgery, National Cancer Institute G. Pascale Foundation (IRCCS), Naples, Italy

**Keywords:** breast cancer, trauma experience, narrative, meaning-making, underfifty women, clinical implications

## Abstract

A diagnosis of breast cancer is considered a potential traumatic event associated with physical and psychological effects. In literature, an exploration of breast cancer experience in young women is lacking, able to shed light on the narrative processes of meaning-making of the experience in specific phases of treatment, as may be the initial impact with the onset of the cancer. Meaning-making processes are determinant aspects when dealing with traumatic events. The research took place at National Cancer Institute Pascale of Naples. We collected 50 *ad hoc* narrative interviews to explore the different domains of the experience with under-fifty women at the first phase of the hospitalization. The Narrative Interviews were analyzed through a qualitative methodology constructed *ad hoc*. Starting from the functions of meaning-making that the narrative mediate we have highlight the different modes to articulate the narrative functions: The Organization of Temporality: chronicled (38%), actualized (26%), suspended (18%), interrupted (16%), and confused (2%). The Search for Meaning: internalized (42%); generalized (24%); externalized (18%); suspended (16%). The Emotional Regulation: disconnected (44%), splitted (28%), pervasive (26%), and connected (2%). The Organization of self-other Relationship: supportive (46%), avoidant (22%), overturned (16%), and sacrificial (16%). The Finding Benefit: revaluating (38%), flattened (34%), and postponed (28%). The Orientation to Action: combative (38%), blocked (36%), and suspended (26%). Findings capture the impact with the onset of the cancer, identifying both risk and resource aspects. The study allows to identify a specific use of narrative device by under-fifty women who impacted with the experience of breast cancer. The ways in which meaning-making functions are articulated highlight the specificity of the first phase of the treatment of the cancer. From a clinical psychology point of view, our findings can be used as clinical narrative markers to grasp, in a diachronic way, the process of meaning-making, integration, and coping during the first phase of breast cancer experience in young women. We consider it valuable to increase longitudinal studies with young women to highlight trajectories of meaning-making during the different phases of the treatment to think about personalized intervention practices diachronically to the experience.

## Introduction

A diagnosis of breast cancer is considered a potential traumatic event associated with physical and psychological effects (Martino et al., 2013, 2015; Villani et al., 2016) that may also occur after the end of medical treatments (Cordova et al., 2007; Elklit and Blum, 2011). Cancer has a specific and peculiar nature because of the difficulty of recognizing a unique stressful. It has internal triggering processes and temporal continuity in terms of hereditary or possible relapse (Gurevich et al., 2002; Mehnert and Koch, 2007).

The threat to life and bodily integrity is often considerable, and the experience of pain, mutilation, and loss of social and occupational roles can trigger overwhelming feelings in a significant minority of affected individuals. The perceived lack of control and impairment imposed by an experience of illness and suddenness of the diagnosis may trigger intense fear, helplessness, or terror, anxiety, depression (Quattropani et al., 2018a,b,c,d).

The psychological trauma, which may occur as a result of such a severely distressing event, in case of cancer starts from the communication of the diagnosis and continue during the different phases of treatment (Martino and Freda, 2016).

Within a socio-constructivist and semiotic perspective, (Salvatore and Freda, 2011; Valsiner, 2014; Salvatore, 2016) the traumatic condition is due to the sudden and unexpected alteration of basic elements governing the relation between women and the external world (Janoff-Bulman, 2004; Joseph and Linley, 2005) and rupture of the temporal continuity resulting in a crisis of meaning processes that support the personal self-narrative of life (Brockmeier, 2000; Neimeyer, 2004, 2006; Martino et al., 2015; Carlino and Margherita, 2016).

The eruption of this experience in the life of young women (under 50 years of age) appears to acquire specific and peculiar characteristics that cannot be overlapped with data present in the literatures, which mainly focused on the target of women over 50 years of age. To date, epidemiological studies bring the attention that in the last 15 years, breast cancer affected 2.4 million women, with the highest incidence in the age group of 34–49 years (Global Burden Disease, 2018) on which a survival rate of 87% is attested.

To date, the clinical psychology perspective still appears to be lacking both in terms of understanding specific characteristics connected to the impact and crossing the experience of breast cancer during a younger age both in terms of proposal of personalized support practices for a target defined as vulnerable by early stages of the therapeutic path (Koopman et al., 2002; Cordova et al., 2007; Chou et al., 2012).

Starting from our previous literature review, we have highlighted some peculiar aspects of this experience.

From a quantitative point of view, research shows that younger women under 50 years live the experience of cancer as a traumatic stressor (Carpenter et al., 1999; Lechner et al., 2003; Manne et al., 2004; Bower et al., 2005; Widows et al., 2005). Young age is a predictor of post-traumatic symptoms higher levels of emotional stress such as depression or anxiety (Wenzel et al., 1999; Broeckel et al., 2000) and a low quality of life following treatment (Baucom et al., 2006). However, functional coping strategies are also identified at a younger age (Manuel et al., 2007; Ali et al., 2014). The fear of recurrence (of cancer) in young women, which is among the possible sources of a minor psychological adaptation during and after treatment (Ahmad et al., 2015), has reached important clinical levels (Costanzo et al., 2007; Mehnert et al., 2009; Taylor et al., 2011; McGinty et al., 2012; Thewes et al., 2013). Quantitative studies have also investigated concerns related to body image according to the type of surgical treatment received (Rosenberg et al., 2013) and co-occurrence with sexual functions (Fobair et al., 2006; Pinto, 2013). In addition, a significant role is played by the gender in cancer patients in particular during chemotherapy that can influence negative emotions (Quattropani et al., 2016).

Few qualitative studies have discussed, in psychosocial terms, the most delicate areas of a breast cancer experience at a young age when women have to deal with a continuous balance between her roles as a working woman, wife, and mother (Adams et al., 2011; Martino et al., 2019).

In a young woman with breast cancer, the role of dependent children’s mother is a key aspect: the women have to maintain the their continuity of life within family context (Coyne and Borbasi, 2006; Ahmad et al., 2015).

Regarding the specificity of breast cancer in young age, concerns are connected to menstrual changes, potential infertility, decisions on pregnancy, breastfeeding, and contraception. Information on the aforementioned concerns needs to be adapted to their age (Dunn and Steginga, 2000; Connell et al., 2006; Peate et al., 2009; Knobf, 2011).

Finally, vulnerability to BRCA 1 and 2 genetic mutations is a relevant aspect at a young age, which opens the need to evaluate important prophylactic medical choices (Rosenberg et al., 2013). In the literature, from a narrative and semiotic point of view, an exploration of breast cancer experience in young women is lacking, able to shed light on the narrative processes of meaning-making of the experience in specific phases of therapeutic pathway, as may be the initial impact with the onset of the cancer. Meaning-making processes are determinant aspects when dealing with traumatic events (Gillies and Neimeyer, 2006; Hall, 2011; Waters et al., 2013; Freda and Martino, 2015) and can explain health outcomes and symptom reduction (Tolstikova et al., 2005; Park et al., 2008). These processes are essential for the adaptation, integration of trauma, and development of well-being (Davis et al., 2000).

### Our Point of View: Narrative and Meaning-Making Process

Within a socio-constructivist and semiotic perspective, we interpreted the narrative device as a space of meaning-making about a traumatic experience (Angus and McLeod, 2004; Hermans and DiMaggio, 2004; Neimeyer, 2004, 2006; Salvatore and Freda, 2011; Valsiner, 2014; Salvatore, 2016) through which the narrator reconstructs a broken self-narrative.

Narration acquires the role of a semiotic device through the narration of traumatic experience and is actualized in the *here and now* of the narrative setting. Through plot development in the narrative process, the narrator sets up processes of semiotic connection, organization, and searching that can promote transformation because he or she tries to find a configuration for events in the discourse that can make sense of the experience, even if temporarily, and thus promote to fill the gap between continuity and discontinuity of the experience (Greenberg and Paivio, 2003; Neimeyer, 2004, 2006; Freda, 2011; Margherita et al., 2015; Dicé and Zoena, 2017). At the same time, the narrative device helps explore the subjective experience and promotes transformation of the experience (McAdams, 2008; Esposito et al., 2017; Margherita et al., 2017). Thus, the device responds, in a natural way (Bruner, 1990; McAdams, 2008), to a fundamental need of the human being: to make experience of a sense of continuity and coherence of the self through the construction of stories in a specific intersubjective and cultural context (Bruner, 1990; De Luca Picione et al., 2017, 2018; Margherita and Gargiulo, 2018) reorganize and construct a form, make the future pre-figurable, and reposition one’s own identity following the onset of the diagnosis. The narrative process is an important mode of the functioning of the mind, allowing the organization and connection of different elements of the experience (such as time, space, behavior, relationships, and actions; Freda and Martino, 2015).

The narrative is an elective tool to promote meaning-making processes that are activated by traumatic life experiences that suddenly confront the person with a new information regarding the world—information that defies the person’s preexisting mental schemas and threatens one’s basic assumptions about the self and/or the world (Horowitz, 1986; Janoff-Bulman, 1992). To study the meaning-making process, we need to observe how people make sense of change and in particular, their efforts in bridging the gap between the appraised meaning and global beliefs and goals (Joseph and Linley, 2005). They can either change their view of the traumatic event to adapt it to their current worldview or change their world view to integrate the traumatic event into global and situational meanings (Janoff-Bulman, 2004). People can make sense of an event in various ways to make it less threatening and more easily managed (Park and Blumberg, 2002; Waters et al., 2013).

Starting from the narrative literature and our previous study using narrative device in the context of illness (De Luca Picione et al., 2017, 2018) we highlight theoretically that the narrative plays the transformative functions of experience. Here we decline such transformations as narrative meaning-making functions that, in a transversal way, allow the development of semiotic articulation that support the processes of adaptation and articulation of the traumatic experience:

#### Organization of Temporality

This refers to connections and insertion of elements of the story within a spatial and temporal framework. It allows a person to organize cancer experience by subjectively connecting different temporal plans of the experience; the narrative allows taking together, in a tridimensional way (past–present–future), the different lines of time (Brockmeier, 2000; Freda et al., 2015).

#### Search for Meaning

This refers to the expression of a person’s need to find order in the world in the face of a traumatic event by looking for meanings that make the event manageable, thereby rebuilding shattered assumptive worldviews and constructing a bridge of sense between continuity and discontinuity (Janoff-Bulman, 1992, 2004; Davis et al., 2000; Bonanno et al., 2004). This function not only refers to finding a meaning in the world but also refers to an engagement in the process of searching and answering the need to know “Why to me?”

#### Emotional Regulation

This refers to the narration process as a dynamic multilevel system that can regulate the relation between emotion and event. The narrative device allows a process of labeling emotions and realizing the important roles that these emotions play in certain events. The narration process is an opportunity to label the emotions and connect them to events in light of the present.

The connection between emotion and event, mediated by the narrative device, establishes the basis and containment for processes of emotional regulation (Tronick, 2010; Valsiner and De Luca Picione, 2017) and meta-reflect on the new version of the experience.

#### Organization of Self-Other Relationship

This refers to the organization of the relationship between the self and the world, between oneself and external worlds, and between oneself and others. The narrative becomes a device of sharing with others, organizing and reorganizing relationship planes in its different aspects and roles (Rimè, 2009).

#### Benefit Finding

This refers to the search for positive aspects for oneself within the traumatic experience and re-evaluation of the onset of cancer; a series of positive changes within the trauma, including an increase in perceived meaning, personal strength, new knowledge, and self-affirmation (Sherman and Cohen, 2006), i.e., positive reflection on a valued self-domain (Tedeschi and Calhoun, 2004).

#### Orientation to Action

This refers to the relation between the narrative construction of experience and ability to make decisions and undertake behaviors and choices that impact events. The narration is, therefore, an open and dynamic process of the construction, regulation, and transformation of its own agency (Brockmeier, 2009; De Luca Picione et al., 2018).

Within a mix-methods longitudinal research design, in this study, we explore different modes in which young women narratively articulate the aforementioned meaning-making functions in first phase of the breast cancer treatment: the hospitalization. This aim allows us to focus our attention on the meaning-making of a vulnerable target during the traumatic experience, an innovative point of view with respect to the literature mainly focused on post-traumatic narrative elaboration, or at the end of the experience. This study gives the opportunity to throw some preliminary clinical reflections on the construction of personalized support interventions for women under 50 with breast cancer and their needs (Villani et al., 2016).

## Materials and Methods

### Participants and Tools

The research was conducted at the National Cancer Institute *“Fondazione G*. *Pascale”*, Naples, which is the national reference for the treatment and care of neoplastic illness, and in the frame of Programme STAR, financially supported by UniNA and *Compagnia di San Paolo*. The research was co-constructed in collaboration with the hospital’s psychology service and breast unit surgery and approved by the medical committee of the National Cancer Institute. The hospital’s psychology service has provided its location and facilities for monitoring meetings and taking charge of women who wanted to continue with the psychotherapeutic support over time.

The women who took part in this research were identified from medical reports and qualified according to the following:

Eligibility criteria: First access to the hospital before the age of 50 years; absence of genetic tests conducted before the onset of the cancer; voluntary participation.

Exclusion criteria: Metastatic disease (stage IV); psychotherapeutic treatments in progress.

The recruitment of women was from January to June 2018. During this recruitment period, all the eligible women, which were booked in the hospitalization institutional lists, invited to participate through a 1-day meeting in the hospital to explain the whole path and aim of the research. We invited 81 women to participate in the research. Of these, 31 have declined. Among the reasons for refusal, we point out the need expressed by the woman to concentrate on the medical procedure, especially if she has to undergo chemotherapy before surgery.

Women’s participation was voluntary, they provided informed consent, and privacy policy was approved by the hospital.

### Narrative *ad hoc* Interview

We constructed an *ad hoc* narrative interview termed as Early Breast Cancer-Processing Trauma Interview (EBC-PTI) for the exploration of meaning-making processes of young women with traumatic breast cancer experience.

The narrative interview comprised nine open questions related to five main areas of the experience of illness. Each question that the researcher keeps in mind without reading it was intended as a narrative prompt able to open a construction of sense following the aforementioned meaning-making functions and shed light on different domains of the experience taking into account its complexity. In fact the interviews were conducted by psychologists, researchers specialized in clinical psychology and with training in the use of narrative devices.

The five main areas of the experience of illness are as follows:

#### Area 1: Story of Experience

This area explores the narration of the illness experience since it appears until the time of the interview. Do you want to try to tell me about your experience of illness starting from when you noticed that something was wrong until now?

#### Area 2: Hypothesis on the Causes of Cancer and Relationships With Similar Experiences

Exploration of natural question related to “Why to me,” search for causes and connection of the present experience to other experiences of the past. Sometimes people imagine why they got sick… do you have any ideas about it or do you imagine anything? Considering how much was shared so far, could you tell me if you have had other experiences that you consider similar to this? How did you deal with them?

#### Area 3: Episodic Deepening

Lunge on an associative, analogical, imaginative level and episodic memory. I would like you to choose three words/adjectives/proverbs or mottoes that come to mind thinking of this phase of experience. Is there an event/episode to which it refers when you say (words X or adjectives X proverbs-mottoes X)? We are interested in knowing what happened, where she was, who else was with her, what she felt, and what she thought at that moment.

#### Area 4: Specific Criticality and Change

Focus on particular scenes about high point of their experience and identifying changes in one’s life. In your personal perspective, do you feel that the experience of illness has changed your way of interpreting life? If so, how? Could you tell me if there is something that has been particularly difficult for you in your journey as of now? Approaching the end of our meeting… Could you tell me if there is anything in particular that you feel you can draw from the crossing of this phase?

#### Area 5: Resources

Area that allows what are the resources and sources of support for women at the specific phase. Could you tell me if there is someone or something that you felt was particularly helpful? How? With whom do you talk to about it?

The questions are placed according to a pre-established order that allows a gradual succession of the traumatic experience, recovering toward the end an anchorage to resources and changes. The interview has an average duration of approximately 45 min and was recorded and then transcribed verbatim.

### Method of Data Analysis

In the first stage of the analysis, we read the narrative interviews, keeping a general question in mind: in what way are the six meaning-making functions articulated in the narratives by women?

We read each narrative interview, searching for each meaning-making process in different modes of narrative articulation. Narrative functions were used as a grid to read narrative interviews. The narratives were treated as one textual corpus.

First, we identified the narrative segment of texts attributable to different narrative functions and then for each identified segment, we attributed an interpretative label that identified the prevalent mode of articulating functions of the meaning-making process. At a later stage, we re-read the modes that emerged from each single meaning-making function and proceeded to an abstraction process to merge similar modes.

At the end of this process, a percentage frequency calculation was performed to observe the presence of modes in all narratives and to highlight the modes prevalently used by women in this phase of their experience in graduality up to the least used ones. Analyses were conducted with the help of three judges who are experts in the qualitative analysis of texts and who individually identified the different modes. The 3 experts coded all the 50 interviews separately and then they discussed and recoded the “labels” according an inter-coder agreement of 98%.

## Results

We recruited 50 women under the age of 50 years (*M* = 42,32; *SD* = 5,35) during the first phase of hospitalization, 40–60 days before breast cancer surgery (Table 1).

**Table 1:** Socio-demographic and clinical characteristics of women.

Variable	All participants (*n* = 50)
Age (years)	42.32(±5.35)
Educational level	
Primary and middle school	22(44)
High school	20(40)
Degree	8(16)
Job position	
Housewife/dis/unemployed	28(56)
Employee Self employed	19(38) 3(6)
Marital Status	
Single	5(10)
Married	37(76)
Separated	6(12)
Widow	1(2)
Number of sons	2(1-2)
Diagnosis Center	
Private	14(28)
Public	33(66)
Not known	3(6)


*Data are reported as number of patients (%), mean (±standard deviations) or median (25th–75th IQR), as appropriate.Numbers of participants with missing data: Marital Status (n = 1).*

In the first phase of breast cancer treatment, the six meaning-making functions were articulated in the narrations of young women according to the following specific modes indicated with percentages of frequencies with which they present themselves.

The *organization of temporality* takes the following modes: chronicled (38%), actualized (26%), suspended (18%), interrupted (16%), and confused (2%).

According to the *chronicled mode*, temporal connections are present between the present and the near past, narrating a time schedule by the medical treatment, ordering the evolution, and the chronological succession of medical tasks and practices. Aspects that precede the *hic et nunc* of narration. This mode highlights a temporal evolution of events, medical examinations, and investigations that have led to the realization that something is critical.

*I noticed a month ago more or less a difference between the two breasts and I decided to go a senological visit, I went to this center where the doctor made me an ultrasound and immediately requested a mammogram and asked me if I wanted to do a needle aspirated. We did these tests and I had already given the possibility that it could be a tumor, from there I moved and I arrived here (id.8)*.

The *actualized mode* highlights a narrative mode according to which in the present time something happens that the woman already knew, that she expected, or to which she felt predestined. The actualization may comprise the pervasion of personal trauma of an already experienced illness that repeats and returns, of the family trauma of a history of breast cancer to which one feels predestined, or previous physiological conditions (fibroadenoma, cysts, etc.) that have been kept under control in a preventive practice and are currently actualized as illness.

*Actually I came for the first time because of a malformation of the tongue and I discovered that it was a benign tumor, and now this ball at the breast… already I lost a sister and in 2010 another sister took her breasts off then, it is not hereditary but it is familiar, as the doctor said (id.24)*.

The *suspended mode* informs that the narration at the present time is still in a condition in which temporality is found on the same horizontal line (past = present = future). Temporal plans of the experience are homogenized in an undefined waiting condition that feeds both doubts and hopes.

*As long as we do not really know what it is, what it is, I do not know what to think, you do all the beautiful and ugly thoughts but in fact you do not know… we do not know yet, we are still in limbo, this is (id.1)*.

The *interrupted mode* shows that in the continuity of life, the disease breaks out as an element of rupture and discontinuity. The temporal horizons are divided at first and after the onset of disease, signaling fractures and fragmentations in one’s ordinary life.

*The date of the report was just the day of my birthday, so it’s like a watershed, up to 40 years everything went well and then started already with another foot (Id.4)*.

Even in a low percentage, there is also the existence of a *confused mode* of organizing time in the narration, signaling the absence of connections related to temporality and a lack of narrative functioning.

*It was prevention, I went to see, because of the money, otherwise I had to pay, but I did it only for two euros, I went through this fact, because otherwise, I did not go (id.42)*.

Regarding the *search for meaning* function, it is articulated narratively according to these modes: internalized (42%); generalized (24%); externalized (18%); suspended (16%).

Within the *internalized mode*, 42% of women search for the meaning of the current experience within themselves. It is allocated in one’s own body, genes, and history and narrated as an attribution of internal causality linked to a body that, with its female functioning (lactation, pregnancies, etc.), is itself the activator of the cancer, or a body that we have not been able to occupy, contributing to making it ill. A body that contains within itself, between fear, guilt, and anger, both pains and experiences (abortion, anguish of death, etc.) never elaborated from a psychic point of view, and a legacy of a disease that seems to pass among female generations of their family, outlining a pervasive history of tumors with which is identified in the *hic et nunc*.

*Maybe because there was my mother, the only idea that comes to me and then… practically more or less at the same age I did the same process more or less… the breast, womb and breast again, so well or bad the same thing, only that she died that she was 41, I ‘m 46-year-old (id.29)*.

Otherwise, the search for meaning function is articulated in narrations also according to the *generalized and externalized* modes. This means, in the first case, that in trying to construct meaning around what is happening, women need to give them a generic meaning, anchoring themselves to the shared/cultural/taken for granted dimensions of meaning that make the world traceable to stable mechanisms that govern it, considering the disease as something that is a part of life and is connected to the whole, without particular causes.

*This is the disease of the century, as if it were an influence at home, in every family there is a case (id.7)*.

In the case of an *externalized mode*, instead we find a narrative process of removing causality from oneself, attributing it to the outside, identifying specific causes in fate, God, in the environment, and in an ungovernable exterior.

*But certainly for what we eat, the air that sucks, this is what brings the disease; do not do something (id.2)*.

Finally, another mode of narration is the *suspended one* in which the women suspended the search for the answer to the question “Why to me?” This is the case that the search for meaning does not seem to come out of the mind and is saturated by the action plan, in which the woman tells how she is focused on the level of doing and of dealing with the situation she has to face.

*“Why did it happen to me? Ah I do not know, it’s not a question I asked myself has arrived, I’m currently facing” (id.1)*.

The *emotional regulation function* is articulated in following modes: disconnected (44%), splitted (28%), pervasive (26%), and connected (2%).

In the *disconnected mode*, they converted all those mechanisms for which emotions that can be linked to the traumatic event must instead be kept away from the consciousness or disconnected from the thought. In this way, we found trajectories of functioning that refer to the confusion, distance, suppression, projection of the representation of affection, and mechanisms narrated by women themselves. There is no clarity on how it feels for them, and it is not yet possible to name the emotions connected to the event. Also, they must be kept hidden so as not to be invaded or have to be taken out of themselves.

*I do not know, I’m in a moment of stasis, i.e*.*, I know it’s me, but it’s like I was not, I’m aware to a certain point, then I leave, as if they were two different persons (id.27)*.

*Actually I do not think about it, that is, as if I were estranged from this thing so as not to make me touch the thing, do you understand? (Id.40)*.

In 28% of the narratives, we find a narrative mode related to the emotions that are *split* in the imaginary. This means that at the acme of the traumatic condition, i.e., at the beginning of the experience of illness, occurs an anticipatory dynamic that places experiences connected to those events; inauspicious experiences that are the most feared by these women; and experiences that are imagined as the most painful and difficult to deal with. A therapy, specifically chemotherapy, opens an imaginary link to the loss of functions typical of the feminine state of health. Also, in the imaginary, appears the post-traumatic condition as a receptacle of a weakness that at the moment cannot exist because it is more important to face the illness.

*Then I thought about chemotherapy, I cannot stand it, it destroys me, I do not want it and I will not accept it, I cannot bear it (id.2)*.

In 26% of the narratives, the narrative function of regulating emotions is organized in a *pervasive mode*. This means that the narrative is configured as the device that can give voice to the emotional specificity of the crossed phase, i.e., to express through flooding emotional reactions the shock linked to the onset of the disease in one’s life.

*I did not expect this diagnosis, my thoughts went to my son (crying), it was a cold shower… I feel angry at myself, I cannot do anything and sadness, so much (id. 43)*.

Finally, a *connected mode* articulates the functioning of emotional regulation, in the sense that an emotion is put at the service of thought that connects various levels of life and allows us to feel emotions. The women meta-reflected on the new connections that are created in the *hic et nunc*.

*This is the second great pain that happens to me in life… I feel responsible for the various choices that can be made, of course I realize that the tumors are a thing still in the phase of discovery, research, so I feel the weight responsibilities and then feel the weight of other things that do not stop in the meantime, the family does not stop, the work does not stop, the house does not stop, I feel less energy for more things to do (id.4)*.

*The Organization of self-other relationship* is articulated in the following modes: supportive (46%), avoidant (22%), overturned (16%), and sacrificial (16%).

According to a *supportive mode*, the areas of support that the woman identifies for herself in this initial phase of illness experience are narratively articulated. The partner, family, and friends are configured as their support, both emotionally and concretely. In particular, other women who have already undergone breast cancer treatment are configured as a source of reflection for their experience. This mode also includes reference to the relationship with the interviewer and the use of the interview context as a possible resource.

My husband was the person who supported me, gave me so much strength, the little girls and my brother, even being male, he can understand me (id 33).

The organization of self-other relationship is articulated in an *avoidant mode* in cases where it is narrated an ambivalence in accepting the closeness of others, preferring moments of solitude, and removing those who try to be present in a way considered intrusive or inadequate. It is not possible to tell anyone what is happening, let alone tell it right away: they can find the right distance, even in terms of time, to share the experience.

*Some people, sometimes not, a bit of intolerance, because you do not even know what you want, sometimes you would like to be alone, sometimes you would like people close but… also depends on the topic of the day… my mom is very apprehensive and does not help me right now (id 3)*.

In the case of an *overturned mode*, the women told of a change in the dynamics, structure, and relational positioning. Above all, within the family, the woman catches a discontinuity in the maintenance of her role, where the concern of the whole system translates into the assumption of different positions that puts the woman in contact with the disease. It is also interesting that in the *overturned mode*, the narration opens up on a woman’s need to take on a different role, receive her care, identify in the illness a test of ties, and understand how reliable and authentic they are.

They do not make me eat more meat, nor more mozzarella, they took away some food in the house that I do not have to touch anymore, because my son is the professor about me, says I do not have to feed my tumor, they check me in the refrigerator, we’re all worried, but we keep it all for ourselves (id 7).

Finally, through a mode that we have defined as *sacrificial*, the narrative organizes a relationship of the woman dedicated to the protection and defense of children and being attentive to the needs and emotions of others, focused on hiding their pain so as not to cause suffering and worry in others.

*I have to hide and show myself to them strong, to them who are my children, my husband, my mother, the sisters, in short, all*. *I have to show that I’m stronger, I’m fine, I’m going to boast and they do not have to worry (id 1).*

The function of *finding benefit* is articulated in the following modes: revaluating (38%), flattened (34%), and postponed (28%).

At the beginning of the onset of cancer, in 38% of the narratives, it is already possible to grasp a *revaluating mode* that articulate on the positive aspect that is believed to help them in the crossing of this experience.

*We are all poor on the face of the earth in the face of these things, I wish I could have more courage in life and overcome, I would like more awareness in my strength (id 44)*.

Instead, the narrative also articulates a *flattened* and a *postponed mode*. In the first case, the woman cannot say what can be the benefits of this experience or if she is connected to an existential continuity, for which nothing has changed yet, or she still does not identify any benefits but starts to grasp negative aspects.

*I do not like how my way of seeing life is changing, I have always had an ideal of how I wanted to be, but apathy, insecurity, envy toward people who are doing well, I do not like this thing, I do not like it the turn that is taking (id 4)*.

According to the *postponed mode*, it is not yet possible to grasp the benefit, growth, or transformative elements at the beginning of the experience, but it is certain that it will be possible to identify them later or at the end of the crossing. This gain naturally comprises the very term of the disease and the resumption of one’s own continuity of life.

*I think my perspective will change, I do not know if it’s already changed now, I think I’ll understand later what I can draw (id 15)*.

The function of *orientation to action* is articulated in the following mode: combative (38%), blocked (36%), and suspended (26%).

In the search for the individual role with respect to one’s condition of illness, strategies for coping are outlined, as in the case of *combative mode*. This means that the woman narrates an active mode in the event in which there is an imperative to fight against it and look for strength within oneself. This mode is oriented to the search for information, relationship with the doctor, and irony.

*I genetically know that I always win, every time I wanted something I always got it, So I want the victory, I want to defeat this thing (id 47)*.

In contrast, the *blocked mode* articulates attitudes based on impotence, mistrust, despair, and resignation and refers to a defenseless position that translates into a dependency positioned toward the medical context. The woman expresses overwhelming feelings and fragility, remaining mostly blocked with respect to the action.

*These days make me feel discouraged, it is poignant, I need to trust, here I feel protected, in the structure, in my city, I have to rely (id 44)*.

Finally, through a *suspended mode*, the narrative highlights the uncertainty related to its positioning at that moment of the experience, in which the possibility to transform the though in action is not yet accessed and not touching the narrative question.

*But no, the awareness is from 10 days so at the moment I do not know (id 34)*.

## Discussion

**FIGURE 1:**
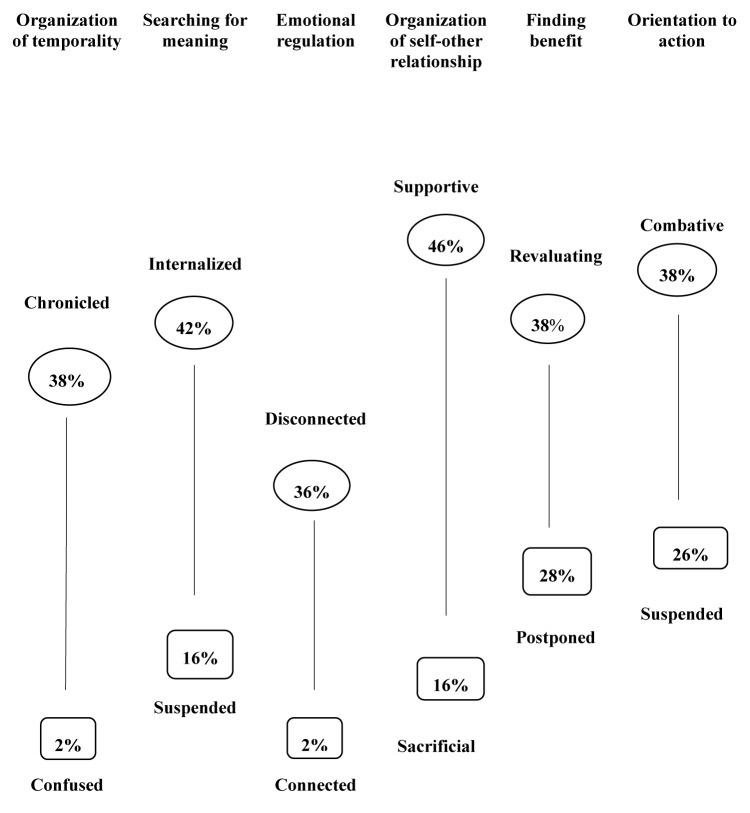
Narrative markers of functions and modes of meaning-making of women under 50 with breast cancer during the first phase of hospitalization.

From a psychological and clinical point of view, Figure 1 sheds light on the possible use of our findings as clinical narrative markers to grasp, in a diachronic way, the process of meaning-making, integration, and coping during the first phase of breast cancer experience in young women. The findings capture the specificity of the impact with the onset of the cancer, identifying both risk and resource aspects useful to reflect about the construction of personalized preventive and supportive settings.

We have illustrated in an initial phase of the onset of a breast cancer how each meaning-making function appropriate for the narrative device is articulated according to the specific mode in the narrations of young women under 50 age.

Making use of a focus aimed at reflecting on modes used by women, to a greater or lesser extent in their narratives, we propose some clinical implications of our study.

Within the function of organization of temporality, women attest on the temporal organization of chronicled mode where their story can be put into words as a succession of events in progress still lacking a temporal frame able to articulate the relationship between the different plans of temporality. This mode appears to be connected in a functional way to the phase of the cancer where the story is narrated, starting from a factual and concrete perspective still in evolutionary construction and which, for now, can be mostly anchored on the present planes. Through this in the *hic et nunc*, the narrative supports, at this stage, a function of putting into words the events crossed by conducting an ordering function of events in their temporal evolution (Freda and Martino, 2015).

On the other hand, a residual number shows a confused mode not yet able to articulate with the planes of time, and therefore, unable to place the story in the space-time of experience.

With regard to the search for meaning, it is mainly articulated within an internalized mode where the possibility of constructing a subjective response to the question “Why to me?” is inscribed in one’s own transgenerational and bodily history, identifying within oneself the responsibilities and causes of illness, specifically, the young age, the inability to take care of themselves and to be more concerned on other areas of their life (job, family, etc.). On the contrary, we find, with a less extent, a suspended mode in which the search for an explanation about what happened is suspended to stay focuses on the action and to cope with the experience in a concrete way (De Luca Picione et al., 2017).

With respect to the emotional regulation function, only a woman can narrate articulating the relationship between emotions and event in order to meta-reflect in the narrative *hic et nunc* on the new meanings of experience. On the other hand, the prevalent mode indicates a time disconnection rather than an emotional regulation and the necessity of disconnecting emotions from the thought and keeping them away. So, we see in an initial phase of impact with the traumatic experience, how there is an interruption of connections between emotions and events and between emotions and thoughts. However, this phenomenon to which we do not give a psychopathological interpretation becomes an indicator of clinical attention in the longitudinal observation of women, e.g., in the crossing of next treatment phases (the surgery, adjuvant treatments, etc.). In this phase, because there is no unblocking of the integration of other information and levels, this mode can be considered as a profound emotional reaction to the shock of the diagnosis and to have a more or less functional role for adapting and coping with the experience and next phases. It will be necessary to pay attention to its narrative evolution toward the ability to give a label on the emotion and differentiate and reflect on emotional states connected to the experience.

The plan of the organization of the relationship between self and others appears to be mainly based on a supportive mode where relationships appear to acquire qualities of support for the phase of illness that the woman is going through when the narration itself is configured as coping tool. In line with literature we find the importance to consider, in a special way, the valuable role on the emotional adjustment to cancer experience played by significant relationships of the women with partners, friends and healthcare professionals (Saita et al., 2015) and the way of manage them during the cancer experience. In a smaller number of cases, relationships also appear to be based on a sacrificial mode where their pain and fragility cannot be shown and contained in the relationship to protect and safeguard it (Margherita et al., 2018). Therefore, it appears also as the burden on women in the maintenance of some relationships to shelter one’s own pain and weakness, as those with young children, which are specific and characteristic areas of women under 50 age.

It appears a resource area to observe how women during this phase of the disease are mainly able to articulate a re-evaluation and the search for a benefit for themselves, to a lesser extent, they evaluate benefits to when medical conditions allow them to feel out of danger.

Also, the relationship with decisions, actions, and behaviors in the narratives appears to be founded on the battle against the disease, where 26% of women suspend the search for their own agentive positioning in the coping of the disease.

We believe that the present study allows us to identify a specific use of narration by young women who are impacted with the traumatic experience of breast cancer in their lives. The ways in which meaning-making functions are articulated highlight the specificity of the first phase of the treatment.

At the purely inferential end, we believe that what emerge from our findings is the expression of physiological aspects related to the disruption of the onset of the cancer in one’s life. It appears that the high presence of disconnected modes in the relationship between emotion and event, the confused mode in the organization of temporality, the suspended mode in the search for meaning, the sacrificial mode in the organization of the relationship between self-other, and the blocked mode in the orientation to actions can represent areas of clinical sensibility on which to promote spaces of containment. If these modes will be maintained by women during the next phases of the cancer treatment as rigid structures unable to synchronize with the variability of the experience, then it could be useful, from a risk prevention perspective, to create spaces and promote a function of putting into words the most fragile emotions where they are kept suppressed, hidden within relationships with children or partner, and promoting a connection with the planes of time. Consistent with the literature, a specific aspect of this experience for young women seems to be precisely connected to this need to stand up and be a guarantor of the continuity of the family system, very often just created (Coyne and Borbasi, 2006; Ahmad et al., 2015).

A need for most women is to experience a secure relationship that can contain the negative emotions generated by the impact of the cancer, treatment, and its consequences. This means being able to think about personalized clinical interventions, meeting spaces designed to experience a relationship of containment and signification of fragility and pain. A space in which the most pervasive emotions find a different narrative space while continuing to hold their own protective role of mother within their own family (Freda and Dicé, 2017). Starting from the synergy with medical staff construct spaces in which give voice to the emotions underlying the waiting and uncertainty times, necessary to the medical world, providing space to the patient to talk and to the explicit expressions of empathy (Mazzi et al., 2013). The authors are aware about the limits of this study linked to the lack of generalizability and the specific context nature of the results, the partiality of the view focalized only on the first phase of hospitalization. Anyway, as this study intends to carry on, we consider it valuable to increase longitudinal studies with young women to highlight trajectories of meaning-making during the different phases of the treatment to think about personalized intervention practices diachronically to the experience.

## Data Availability

The datasets generated for this study are available on request to the corresponding author.

## Ethics Statement

This study was carried out in accordance with the recommendations of ‘name of guidelines, name of committee’ with written informed consent from all subjects. All subjects gave written informed consent in accordance with the Declaration of Helsinki. The protocol was approved by the ‘Ethical Committee of The National Cancer Institute Pascale of Naples with managerial decision of N. 36 del 18/01/2018.’

## Author Contributions

MM is the scientific coordinator of the project, she developed the theoretical framework of the present study, designed the research project, and contributed to the scientific supervision of the entire study. DL and AG contributed to the methodological approach, development of narrative interview, collection of data, and wrote the manuscript. DB, VA, FA, and RT support the construction of the introduction and interpretation of findings. All authors discussed the results, commented the manuscript, and gave the final approval of the work.

## Conflict of Interest Statement

The authors declare that the research was conducted in the absence of any commercial or financial relationships that could be construed as a potential conflict of interest. The reviewer RP declared a shared affiliation, with no collaboration, with several of the authors, MM, DL, and AG, to the handling Editor at the time of review.
